# The Green Tax Revolution

**DOI:** 10.1007/s10272-021-1000-y

**Published:** 2021-10-05

**Authors:** Csaba László

**Affiliations:** Institute of Economics and Public Policy, Corvinus University of Budapest, Fovám tér. 8, 1093 Budapest, Hungary

## Abstract

Climate crisis is becoming higher on the agenda of the decision makers of the world. A huge amount of resources have been dedicated to green projects, however far less emphasis has been put on tax policy opportunities. Carbon pricing can increase the burden of CO_2_ producers, but this does not appear to be enough. We need a Green Tax Reform which focuses on the Pigouvian approach and can correct the distortions of different climate hurting activities. Through tax policy tools, the price structure should be drastically changed and serious incentives should be provided to change the behaviours of the consumers and producers to achieve green policy goals.

*Should we give up hamburgers?* In recent years, increasing emphasis has been placed on the importance of tackling the climate crisis and seeking economic and social policy solutions. We see the scientific community outlining with increasing confidence the scenarios that will fundamentally transform our natural environment within decades, and the significant economic and social impacts of this can barely be assessed yet. Yet one thing is sure: The current economic and social models are unsustainable. Although progress is being made in many areas, we have not reached a critical mass. All the proposals on politicians' tables are not enough yet.

After the outbreak of the COVID-19 pandemic, it was frequently said that this could be a good lesson for humanity, an impetus to transform our way of life. But looking at the actual intentions, it is clear that people want their lives back, politicians want to catch up almost immediately with production and consumption, and the business sector obviously wants a return to the nice pre-crisis profits too. There does not seem to be the emergence of a model change. Make no mistake, many people recognise the need for action and would take serious steps, but they are expecting today's economic and social models to continue. One can be angry at them, but as long as the harmful effects of the climate crisis on voters and consumers do not fundamentally change public attitudes, a radical turnaround cannot be expected.

* The author wishes to thank László Andor and Elemér Terták for their help and support.

Most of the approaches so far have taken the form of a project or regulation, but the possibility of a substantive tax reform at the level of "big politics" has not arisen. Is it advisable to ignore an opportunity like the one the tax system could provide?

## The big picture

In the European Union, the ratio of total tax revenues to GDP ranged from 39.7% to 40.1% between 2015 and 2019 (see Table 1).[1]

To illustrate the sizes, it is worth noting that the total tax revenue of the EU27 in 2019 was €5,595.5 billion, of which €330.6 billion, or 2.4% of GDP, was environmental tax revenue. This is a lot of money, but most of it is actually an excise tax that was collected by states even before anyone talked about environmental protection. In most countries there is no rule for earmarking these revenues for environmental purposes. Transport-related fuel taxes and other transport taxes account for 1.6% of the 2.4% environmental tax burden relative to GDP. It follows that other energy taxes account for 0.6% of GDP, and most strikingly, the share of taxes on pollution and resources is only 0.1% of GDP (€10.6 billion).

If we look at the trends, this approach does not seem to change at EU level either. The ratio of taxes of an environmental nature, taxes on energy, taxes on fuels and taxes on pollution and resources relative to GDP has essentially not changed significantly since 2007 (European Commission, 2020a).

1 In Eurostat's detailed tax statistics, 2019 is the last available year.

A significant portion of the EU 2021-2027 budget will very rightly serve climate goals. In the next seven year period, the EU plans to spend 33% (€356.4 billion) of the Multiannual Financial Framework on natural resources and the environment (European Commission, 2020b). One possible form of repayment for a bond issue to finance the EU rescue package could be the Carbon Border Adjustment Mechanism and the EU Emissions Trading System (European Commission, 2020b). These tools can be useful in achieving climate goals, but they do not have a significant impact on the EU's tax structure, while possible new taxes at the EU level have nothing to do with environmental goals. Tax harmonisation is a particularly sensitive issue in the EU. The adoption of any EU-wide proposal requires the support of all member states, yet we can be sure that some would oppose a green tax reform (GTR). Nonetheless, neither the Commission nor the individual member states seem to have seriously raised the possibility of this type of shift. In European tax policy, the relatively harmonised types of tax include VAT and excise taxes. Although the decline in international energy prices in recent years has helped to curb inflation, those responsible for tax policy missed a huge opportunity at the national and EU levels when price declines were not offset by tax increases. Significant tax revenues could have been generated with consumers not noticing an increase in their burdens, and all the while generating additional revenues either to finance climate goals or to reduce other taxes.[2] The incentivising role of taxes in curtailing emissions would not have diminished either.

## Why are we not talking about a green tax reform?

In the mainstream media, there is rarely a question raised about greening the tax system in relation to climate change. It cannot be said that economics has not dealt with the issue for a long time.[3] Yet for at least 30 years, the profession has also been studying the impact of a tax reform where the tax system's focus would shift to environmental taxes, with a parallel reduction in labour taxes. Not only could this transformation have the advantage of sending clear price incentive signals to the business sector and society to reduce their harmful activities, but reducing taxes on labour is likely to improve the employment situation too. The European Environment Agency (EEA, 2011) examined 26 studies and Maxim and Zander (2019) examined 33 studies on this matter. While clearly much depends on individual country situations, generally speaking these studies have displayed positive employment effects.

2 This effect is called the double dividend approach in economics.
3 The concept of externalities developed by Pigou was addressed in his book published back in 1920. The possibility of adjusting with the so-called Pigovian corrective taxes, which is one of the solutions to undesirable externalities, has long been part of universities' introductory courses to economics.

Table 1
**Tax revenue in the EU27, 2015-2019**
in % of GDP

| Main tax aggregates | 2015 | 2016 | 2017 | 2018 | 2019 |
|---|---|---|---|---|---|
| Total taxes (including compulsory actual social contributions) | 39.7 | 39.8 | 39.9 | 40.1 | 40.1 |
| Direct taxes | 12.9 | 13.0 | 13.2 | 13.2 | 13.3 |
| Taxes on individual or household income including holding gains | 9.4 | 9.3 | 9.4 | 9.5 | 9.6 |
| Taxes on the income or profits of corporations including holding gains | 2.4 | 2.6 | 2.7 | 2.7 | 2.7 |
| Taxes on property | 2.3 | 2.3 | 2.3 | 2.2 | 2.2 |
| Indirect taxes | 13.7 | 13.6 | 13.6 | 13.7 | 13.7 |
| Environmental taxes | 2.4 | 2.5 | 2.4 | 2.4 | 2.4 |
| Taxes on energy | 1.9 | 1.9 | 1.9 | 1.9 | 1.8 |
| Taxes on energy, of which transport fuel taxes | 1.3 | 1.3 | 1.3 | 1.2 | 1.2 |
| Transport taxes (excluding fuel taxes) | 0.5 | 0.5 | 0.5 | 0.5 | 0.4 |
| Taxes on pollution and resources | 0.1 | 0.1 | 0.1 | 0.1 | 0.1 |
| Total actual compulsory social contributions | 13.1 | 13.2 | 13.1 | 13.2 | 13.1 |

Source: European Commission (2020a).

So why does it barely reach politicians and the public that taxation could also help with the climate crisis? One of the relatively few publications of this type is Odendahl's (2020) article entitled "The Green Tax Revolution Europe Needs". He is not the only one to state in this matter that we have a relatively clear and simple solution before our eyes (see EEA, 2011). His suggestions can be summarised as follows:

- carbon emissions must be taxed, including the sectors that have been left out so far (construction, transport, agriculture);
- subsidies that surprisingly still exist in industries that are significant emitters of $CO_2$ must be eliminated;
- taxes on labour should be reduced using the extra revenues generated in this way;
- finally, Odendahl rightly adds that the realignment of price ratios can understandably lead to serious losses for economic and social groups, so the extra revenue must be used to help the adaptation of those who lose out.

If it is that simple, why has it not been done? There might be several different reasons why GTR has been pushed so much into the background. There is a prominent recent example. The yellow vest protests in France were not likely to whet politicians' appetite for a drastic increase in fuel taxes. One of the main risks of any such reform is that identifying the losers and compensating them at any level is already a very difficult administrative task. This means that the losers price their own losses fairly quickly and have little interest in the fate of the planet or the destruction of the Amazon rainforest. However, if environmental protection would make their lives difficult, they would say "no thank you". Politically, therefore, it is very risky to upset loud and influential groups.[4]

Another problem is that there are many other important and useful initiatives in this matter. Governments can use regulatory tools, for example to reduce vehicle emission standards. Separate state subsidies could be linked to the introduction of green technologies.[5] Every day we read interesting articles about what kind of new technological marvel is on the horizon. Without completely underestimating the impact of any electric car or solar collector, these steps may give the impression that there is an easy solution as well. Without going too deeply into the details, it appears that the known measures and technologies cannot guarantee the outcomes that politicians dare to promise. In the meantime, how much more comfortable it is to live a consumer society life and to trust in the miraculous effects of new technologies.

However, we might not be that lucky. One of the fundamental problems is that only one technology with a reasonable cost is currently known to sequestrate large amounts of $CO_2$: afforestation. If humanity could stop emitting $CO_2$ from one moment to the next, there would probably still be too much $CO_2$ in the air, and this process would only be exacerbated if – due to the current level of warming – significant amounts of methane were released, a much larger greenhouse gas than $CO_2$, which has virtually been sequestrated frozen in the northern tundra in recent millennia.

## So what is next?

The reasons why GTR has not been on the agenda, despite the fact that this approach is also characterised by a clear concept and promising positive side effects, are outlined above. Nevertheless, modern humanity does not enjoy such a privilege as to reject this opportunity. Moreover, the more radical reform we talk about, the greater structural changes we have to prepare for, the more unforeseen side effects we can expect, and the more protests we can anticipate from losers who fare badly in their own daily lives. For all these reasons, no matter how little time there is, two important principles must certainly be laid down.

### International cooperation

At today's level of integration in the international economy, perhaps only the largest countries or economic communities can afford to embark on a completely new path in terms of taxation, but even for these countries it poses many risks if they alone put their entire tax system at the service of the climate. Moreover, to reach a critical mass on a global scale, well over 50% of the earth's economy and population must shift to a completely different environmental footprint relatively soon. GTR must therefore be based on international cooperation and harmonisation. It will not be easy: In the EU, in the case of taxation, only one single country is needed to block any proposal.

### Gradation

The other important principle is gradation. Due to the complexity of the topic, there is a good chance that even with the most careful preparation there will be numerous negative side effects that need time to be mitigated. In their study, Koskela and Schöb (1999) highlighted the possibility, for example, that a simultaneous increase in the carbon tax and a reduction in the tax burden on labour could lead to an increase in the consumption of the products intended to be reduced. After all, if the loss of income stemming from an increase in the price of those products is overcompensated by the income surplus of a personal income tax reduction in social groups where the consumption is concentrated, the "weapon may backfire" and the consumption of the given product, and thus harmful emissions, may even increase.

In one of the best summaries on the subject, Groothuis (2018) refers to The Ex'tax Project, according to which there are more than 100 different tax base options available to governments. So it is evident that a truly comprehensive GTR would probably be the most complex economic policy intervention in economic and social life in the last 100 years; but caution and gradation are certainly justified.

## The radical approach

While a number of aspects have been listed so far as to why a comprehensive and radical approach is not making progress, we cannot dismiss the need for a radical GTR. When it comes to acceptance, politicians always tend to blame poor communication for the failure of their own far-reaching reforms. In the world of fake news, it may be na-

4 In one of the world's largest economies, the USA, it is virtually a surefire path to political failure if a candidate talks about raising petrol taxes during an election. Yet if there is anywhere that should have doubled or tripled petrol taxes decades ago, it is the USA. The average consumption of the US car fleet could have been much lower, and more people would also use more advanced public transportation.

5 Scepticism about the effectiveness of regulations is perhaps understandable in Europe after the VW diesel scandal. Another regulatory failure seems to be the support of plug-in hybrid vehicles in several countries.

ive to rely on communication resting on common sense, but unfortunately, we do not really have any other choice. Yet the vivid images of extreme weather and natural disasters that have multiplied in recent years may help the public and therefore politicians to change their minds about the radical action that is required. We definitely need to go further than the usual recipe to tax only direct $CO_2$ emissions. All areas that have a significant, even if indirect, impact on greenhouse gas emissions should be taxed. The more than 100 tax base options previously mentioned need to be looked at one by one.

Another approach that has been neglected must also be brought to the fore. If it is true that afforestation is currently the only $CO_2$ sequestrating technology that also works on an "industrial" scale, then humanity's use of land will need to be fundamentally transformed too. Perhaps we need to go back to the comprehensive use of the oldest type of tax: land tax. Will Georgism inspired by Henry George in the 19th century make a return? With the concept of the "single tax", George and his followers wanted to rely mostly on land tax for state revenues.[6] In the 21st century, a differentiated land tax could be levied depending on the nature of land use, and with today's satellite and drone technology, it would also be relatively easy to control.

With current technology there are basically three ways to significantly reduce land use.

First, improve technology efficiency. This is what thousands of scientists and business people are constantly working on. Market competition is doing its job in this area.

Second, reduce food waste. Estimates of global food waste are staggering, and suggest, incidentally, that food is already so cheap for a significant proportion of consumers that they do not care about thrift. So, let us raise the tax on food (while there is barely any country that does not have preferential taxation on food production or consumption). We are treading on thin ice here because even in the richest societies, food prices are really a matter of daily survival for the poorest people. Not to mention the poorest countries, where it is not the most impoverished 5%-10% but even 50%-60% of society that can be affected by the price of basic food. Many politicians and dictators could tell us how rising food prices led to the loss of their power after the outbreak of food riots.

Third, base food taxation on the intensity of land use. This may seem a heretical idea, but the same caloric intake can involve up to more than ten times the land requirement depending on what foods we partake. The consumption of food of animal origin, mainly cattle, can require land use more than ten times that of plant-based foods. Differentiated taxation of food should be introduced depending on the land requirements for their production.

There is little doubt that proposals of this kind may continue to be held in derision for some time, yet if the structure of human consumption were to shift from meat, especially beef, to plant-based foods, huge areas of land could be freed up for the replanting of former native vegetation. This in turn would offer a chance for the 21st century to avoid a great wave of animal and plant extinction. By way of digression, it is worth noting that morbid obesity, which is present in a significant part of the world and is becoming a public disease, requires a proper solution not only because of the indisputable health problems associated with it. The role of physical exercise and a healthy lifestyle in reducing obesity is undeniable, but lower calorie intake and a healthier diet would certainly result in less food consumption, and less agricultural land use.

## Closing remarks

If humanity fails to find a solution to the climate crisis, we will not be able to talk about lasting and sustainable economic growth in the coming decades. Tax policy should play a much bigger role in this fight. It is not an easy task, but we might as well get used to the taste of green taxation. Technical constraints are unlikely to prevent the reform of the tax system. Known techniques of turnover taxation are essentially used widely across the globe, and land tax was even collected in ancient Mesopotamia. We are at the beginning of a long journey.

6 The great attraction of the single tax was that it was almost impossible to defraud the land tax imposed on the landowner.
